# Statistical Analysis of Synthesis Parameters to Fabricate PVDF/PVP/TiO_2_ Membranes via Phase-Inversion with Enhanced Filtration Performance and Photocatalytic Properties

**DOI:** 10.3390/polym14010113

**Published:** 2021-12-29

**Authors:** Erika Nascimben Santos, Ákos Fazekas, Cecilia Hodúr, Zsuzsanna László, Sándor Beszédes, Daniele Scheres Firak, Tamás Gyulavári, Klára Hernádi, Gangasalam Arthanareeswaran, Gábor Veréb

**Affiliations:** 1 Department of Biosystems Engineering, Faculty of Engineering, University of Szeged, Moszkvai Blvd. 9, HU-6725 Szeged, Hungary; erikansantos@outlook.com (E.N.S.); fazekas@mk.u-szeged.hu (Á.F.); hodur@mk.u-szeged.hu (C.H.); zsizsu@mk.u-szeged.hu (Z.L.); beszedes@mk.u-szeged.hu (S.B.); 2 Doctoral School of Environmental Sciences, University of Szeged, Dugonics Square 13, HU-6720 Szeged, Hungary; dani.firak@gmail.com; 3 Department of Inorganic and Analytical Chemistry, Institute of Chemistry, University of Szeged, Dóm Square 7, HU-6720 Szeged, Hungary; 4 Department of Applied and Environmental Chemistry, Institute of Chemistry, University of Szeged, Rerrich Béla Sq. 1, HU-6720 Szeged, Hungary; gyulavarit@chem.u-szeged.hu (T.G.); hernadi@chem.u-szeged.hu (K.H.); 5 Institute of Physical Metallurgy, Metal Forming and Nanotechnology, University of Miskolc, Miskolc-Egyetemváros, C/1 108, HU-3515 Miskolc, Hungary; 6 Membrane Research Laboratory, Department of Chemical Engineering, National Institute of Technology, Tiruchirappalli 620015, Tamilnadu, India; arthannareeg@nitt.edu

**Keywords:** polyvinylpyrrolidone, photocatalytic membrane, TiO_2_ nanoparticles, statistical analysis, central composite design

## Abstract

Non-solvent induced phase-inversion is one of the most used methods to fabricate membranes. However, there are only a few studies supported by statistical analysis on how the different fabrication conditions affect the formation and performance of membranes. In this paper, a central composite design was employed to analyze how different fabrication conditions affect the pure water flux, pore size, and photocatalytic activity of polyvinylidene fluoride (PVDF) membranes. Polyvinylpyrrolidone (PVP) was used to form pores, and titanium dioxide (TiO_2_) to ensure the photocatalytic activity of the membranes. The studied bath temperatures (15 to 25 °C) and evaporation times (0 to 60 s) did not significantly affect the pore size and pure water flux of the membranes. The concentration of PVDF (12.5 to 17.5%) affected the viscosity, formation capability, and pore sizes. PVDF at high concentrations resulted in membranes with small pore sizes. PVP affected the pore size and should be used to a limited extent to avoid possible hole formation. TiO_2_ contents were responsible for the decolorization of a methyl orange solution (10^−5^ M) up to 90% over the period studied (30 h). A higher content of TiO_2_ did not increase the decolorization rate. Acidic conditions increased the photocatalytic activity of the TiO_2_-membranes.

## 1. Introduction

Membrane filtration is widely used for wastewater treatment since it has numerous advantages such as low cost, low energy requirement, high removal efficiency, and that no chemical additives are necessary for it [1,2].

One of the most popular methods for membrane fabrication is phase-inversion, especially the non-solvent induced phase separation method. It involves adding a casting thin film of a polymer/solvent solution (liquid state) to a non-solvent bath after which the solvent/non-solvent exchange occurs at a certain demixing rate. As a result, the liquid is converted to a solid-state thin film with pores, which is the membrane itself [3]. Phase-inversion is a very complex process in which both thermodynamic and kinetic parameters are important, making it difficult to predict its course. It is essential to understand not only the factors influencing the performance of membranes but the synthesis process as well, in order to be able to set proper, specific parameters during their fabrication, since even small changes in one parameter can drastically influence the characteristics of a membrane [4]. Various conditions affect the demixing rates during fabrication and, therefore, the formation of membrane pores. The membrane pores can be controlled in several fabrication steps, for instance: altering the composition of the dope solution (type and concentration of polymer, solvent, and additives), the casting conditions (temperature, thickness, evaporation time, support material), and the precipitation conditions (type of non-solvent, time, temperature) [4,5].

Polyvinylidene fluoride (PVDF) has been widely used to fabricate membranes since it has a high chemical, thermal, oxidation, and UV stability [6,7,8,9,10]. Despite their advantages, PVDF membranes have a hydrophobic nature, causing a delay in the solvent/non-solvent exchange during phase-inversion preparation, resulting in a dense membrane with very low flux [11]. To address this issue, several studies have already investigated the enhancement of membranes by varying functional groups, combining polymers, adjusting cross-link density, including additives, etc. [12]. Additives can be included in the membrane matrix via different methods, such as: (i) coating and grafting, in which the membrane is modified after its fabrication; and (ii) blending, which includes the addition of the given material in the matrix during the fabrication process [6,13,14]. The functionalization of membranes can enhance the immobilization of additives, thereby enhancing their stability and properties [15,16,17,18].

The addition of materials can increase the permeability, selectivity, flux, and anti-fouling properties of PVDF membranes [6,19,20,21]. Hydrophilic polymers such as polyvinylpyrrolidone (PVP) [10,14,15] and polyethylene glycol (PEG) [22,23] are widely used for the enhancement of the membrane, as well as hydrophilic nanoparticles, for instance, such as silicon dioxide (SiO_2_) [24], zinc oxide (ZnO) [19], and titanium dioxide (TiO_2_) [21,25]. Using nanoparticles with photocatalytic properties can also result in self-cleaning membranes when activated by light [14,26]. TiO_2_ is the most investigated photocatalytic nanoparticle due to its high chemical stability, photocatalytic activity, availability, and low cost [8,18,27,28,29].

Attention should be drawn to the various effects of additives, since their concentration and type can either limit or enhance the solvent/non-solvent demixing rates and therefore, the formation of macrovoids, which can be beneficial depending on the membrane requirement [30]. Instantaneous demixing rates generate membranes with macrovoids in a finger-like structure, while delayed rates are responsible for forming membranes with a sponge-like structure. These macrovoids results in thin membranes that can be advantageous for micro- and ultrafiltration, efficiently separating the compounds with a viable flux at low pressure. However, these thin membranes also have low mechanical strength and are prone to compaction, which are not suitable for some other high-pressure applications, such as gas separation, nanofiltration, and reverse osmosis [4,5,31].

Despite the extensive use of PVDF for membrane fabrication, there is a huge discrepancy in the literature regarding the conditions of phase-inversion. Many studies have investigated different conditions separately, varying one factor while keeping the others fixed [32,33,34,35]. Recently, a few researchers have started to evaluate the variable conditions simultaneously, for which statistical analysis is required [36,37,38,39,40,41]. As a result, the statistical approach for membrane fabrication using the response surface methodology became an important step. This method can evaluate the effects of all possible combinations of different factors individually or when they are interacting with each other; therefore, membranes with defined characteristics can be fabricated.

Zhao et al. [39] performed a statistical analysis to evaluate the performance of membranes for filtering yeast dispersions in a series of experiments where they used three different solvents and different PVDF, PVP, and graphene-oxide contents. Back et al. [37] studied how the molecular weight of polymers (PVDF and PVP) affects the dextran removal efficiency of membranes using various polymer concentrations, bath and dope solution temperatures. Ahmad et al. [36] investigated how the bovine serum albumin protein (BSA) binds to the membrane surface when altering the PVDF concentration, dope solution temperature, and casting thickness. Orooji et al. [38] fabricated membranes via phase-inversion using different solvent ratios, evaporation times, and bath compositions to improve pure water flux and remove BSA from water. Ghandashtani et al. [41] fabricated various microfiltration membranes to treat oil-in-water emulsions and evaluated the effect of three different solvents while varying the evaporation time, relative humidity, and concentration of SiO_2_ additive. Vatanpour et al. [42] investigated the different PVDF and TiO_2_ contents of UF membranes while varying operational conditions of submerged photocatalytic membrane reactors to evaluate the photocatalytic decomposition of Rhodamine B dye. Several other authors performed statistical analysis to optimize the operation conditions of filtration instead of the fabrication conditions of membranes [40,43,44]. It is important to highlight that several factors can be taken into consideration when choosing the fabrication conditions of membranes; however, the addition of more variables to a statistic model considerably increases the extent, progress, and complexity of experiments and the understanding of outcomes.

To the best of our knowledge, there is no work published in which statistical analysis is performed at various fabrication conditions for neat, PVP-, and TiO_2_-modified PVDF UF/MF membranes focusing on pure water flux, pore size, and photocatalytic characteristics. In this study, a central composite design (CCD) was used to screen the variables affecting the fabrication of PVDF membranes. The effects of coagulation bath temperature, evaporation time, contents of PVDF, PVP, and TiO_2_ were investigated on the membranes. The pure water flux, pore size, and photocatalytic activity of the membranes were evaluated to determine intervals of fabrication and conditions under which membranes with specific attributes can be produced.

## 2. Materials and Methods

### 2.1. Fabrication of Neat Membranes

The membranes were fabricated using phase inversion with the immersion precipitation method. First, calculated amounts of polyvinylidene fluoride (PVDF, 64.03 kDa, Alfa Aesar^TM^, Kandel, Germany) and polyvinylpyrrolidone (PVP, 40 kDa Sigma-Aldrich^®^, Saint Louis, MO, USA) polymers were dried in an oven at 80 °C for 4 h. Then, 20 g of dope solution was fabricated following the conditions presented in Table 1 in the following sequence: different mixtures of dried PVP (0.0, 2.5, and 5.0 wt%) and PVDF (12.5, 15.0, and 17.5 wt%) were added to a glass vessel followed by the addition of the solvent N-methyl-2-pyrrolidone (NMP; 99.13 kDa, 1.028 g mL^−1^ density, Molar Chemicals Kft., Halásztelek, Hungary). The solutions were stirred for 20 h at 20 rpm and 60 °C, followed by 30 min of ultrasonication (UP200S, Hielscher, Teltow, Germany) at maximal amplitude. After that, the solution was aged for 24 h in the dark at the same temperature to assure the total removal of air bubbles formed during the previous 20-h-long stirring. To determine the most suitable temperature for the fabrication, preliminary experiments were carried out at different temperatures (25, 40, 60, and 80 °C). 60 °C was found to be the best temperature because the membrane presented the best formation with good viscosity for casting. This result is in good agreement with the literature [24,45].

The dope solutions were set aside until room temperature was reached, and then they were cast on a glass plate using a casting knife (BGD205 type, 160 mm wide, Biuged Laboratory Instruments Ltd., Guangzhou, China) at 200 μm thickness. The partial solvent/non-solvent exchange was done by setting aside the glass plate for different evaporation times (0, 30, and 60 s). Then, the glass plate was immersed in a non-solvent bath containing ultrapure water (PureLab chorus, ELGA, Veolia, Celle, Germany) and 3 g L^−1^ sodium lauryl sulfate surfactant (97.8%, Molar Chemicals Kft., Halásztelek, Hungary) at different bath temperatures (15, 20, and 25 °C) for 60 min. These parameters are consistent with most of the papers published on this topic. [22,24,37,46,47,48,49,50,51,52,53,54]. After that, the membrane was washed with ultrapure water and kept for 48 h in an ultrapure water bath at room temperature prior to the experiments. The surfactant was used to reduce surface tension at the interface of the polymer and the non-solvent bath, and to assure the formation of a membrane with a porous structure, which were observed and mentioned in previous studies [9,22,23,55,56].

To evaluate how the fabrication conditions influence the membrane performance, four different variables with low (−1) and high (+1) levels were analyzed by CCD (Table 1). The chosen values are within the interval that is most commonly applied for the preparation of membranes [37,38,40]. It is important to highlight that using values beyond these limits could result in membranes with too high or too low viscosities that could not be cast properly. Another possible drawback could be the formation of membranes with extremely fast demixing rates, thus with too many holes or hardly any porosity.

The total number of experiments was 27 (2^4^ different levels, eight face-centered star-points, and three center points to retain the curvature). All the membranes were fabricated in random order. The statistical analysis was carried out using Statistica^TM^ Software (version 14.0.0.15, TIBCO Software Inc., Palo Alto, CA, USA). The model validation was done using analysis of variance (ANOVA) with a significance level of 0.05 (*p*-value < 0.05) [37,38,42,44]. Three-dimensional response surfaces were generated using the independent variables (bath temperature, evaporation time, concentrations of PVDF, and PVP) and responses (pure water flux and pore size).

### 2.2. Fabrication of TiO_2_-Modified Membranes

The optimization of the fabrication of TiO_2_-modified membrane was required to obtain membranes with high flux and high photocatalytic activity using the same CCD methodology. Commercial titanium dioxide (TiO_2_; Aeroxide P25, containing 90 wt% anatase and 10 wt% rutile; D_anatase_ = 25.4 nm and D_rutile_ = 40 nm, Evonik Industries, Hanau-Wolfgang, Germany) was added to the dope solution in different concentrations (0, 1.5, and 3.0 wt%) to investigate the optimal ratio of nanoparticles that could improve the flux and ensure that the membrane had significant photocatalytic activity. First, the nanoparticles were dried in an oven at 80 °C for four hours. Then, a calculated amount of TiO_2_ was added to the NMP solvent, and the suspension was ultrasonicated for two minutes (UP200S, Hielscher, Teltow, Germany). After that, the additive (PVP) and polymer (PVDF) were added, and the same fabrication method detailed above was carried out.

The chosen factors were the concentrations of PVDF, PVP, and TiO_2_, and their values were chosen based on the results of the previous optimization of the neat membrane (Table 2). To fabricate homogeneous casting solutions, the maximum values of PVP and TiO_2_ were set to 3%. The photocatalyst content used in this study is in good agreement with that used by several other authors [21,40,57].

Since three variables were considered for the modified membrane, the total number of experiments was 17 (2^3^ different levels, six face-centered star points, and three center points). The membranes were fabricated in randomized experiments.

### 2.3. Membrane Characterization

#### 2.3.1. Filtration

The membranes were kept wet in a water bath at room temperature until the beginning of the characterization experiments. Initially, pure water was filtered in a batch-stirred dead-end membrane reactor (Millipore XFUF07601, Burlington, MA, USA) which was equipped with the given wet membrane (active filtration area: 36.2 cm^2^). The water was filtered by applying a transmembrane pressure of 100 kPa and stirring speed of 250 rpm for 30 min to reach a steady state and guarantee the compaction effect. Then, the flux was recorded with a computer-controlled scale at predetermined intervals to obtain the pure water flux (PWF).

The fluxes (J) were calculated as follows:

(1) $$J=\frac{\Delta V}{A\;\Delta t}$$

where J is the permeation flux (L m^−2^ h^−1^), ΔV is the permeate volume (L), A is the effective membrane area (m^2^), and Δt is the sampling time interval (h).

#### 2.3.2. Morphology

The porosity (ε) of a membrane shows the ratio of pore volumes (wet volume) and the total membrane volume, which was calculated as follows:

(2) $$\varepsilon =\frac{\frac{m_{\mathrm{wet}}-m_{\mathrm{dry}}}{\rho_{w}}}{\frac{m_{\mathrm{wet}}-m_{\mathrm{dry}}}{\rho_{w}}+m_{\mathrm{dry}}\left(\frac{\%\mathrm{PVDF}}{\rho_{\mathrm{PVDF}}}+\frac{\%\mathrm{PVP}}{\rho_{\mathrm{PVP}}}+\frac{{\%\mathrm{TiO}}_{2}}{\rho_{{\mathrm{TiO}}_{2}}}\right)}$$

where m_wet_ and m_dry_ are the masses of wet and dry membranes (g), respectively; ρ_w_, ρ_PVDF,_ ρ_PVP_, and ρ_TiO_2__ are the densities of water, PVDF, PVP, and TiO_2_ (997, 1780, 1200, and 4260 kg m^−3^, respectively); %PVDF, %PVP, and %TiO_2_ are the weight percentages of the materials that were used to fabricate the membranes. After the membrane fabrication, pieces of wet membranes were soaked in water for 48 h, then the excess water was carefully removed with a tissue to determine the wet weights. After that, the pieces were dried for 24 h at room temperature to determine the dry weights.

The Guerout-Elford-Ferry equation [39,54,55,58,59,60,61] was used to calculate the mean pore radius of the membranes (r), and the results were multiplied by two to calculate the membrane pore sizes (diameter, d = 2r):

(3) $$r={\left(\left(2.9-1.75\;\varepsilon \right)\times \frac{8{\;\eta }_{W}\;l\;J}{\varepsilon \;\Delta P}\right)}^{0.5}$$

where ε is the calculated porosity, η_w_ is the viscosity of water at room temperature (0.00089 Pa s), ΔP is the operation pressure (Pa), J is the measured pure water permeation flux (m^3^ m^−2^ s^−1^), and l is the membrane thickness (m).

The morphology of some fabricated membranes was also characterized by field emission scanning electron microscopy (FESEM, Hitachi S-4700 Type II, Krefeld, Germany) on the cross-section and top surface. The applied acceleration voltage was 10 kV. The membranes were dried and then broken after cooling with liquid nitrogen (making it possible to take cross-sectional images) and were coated with gold before analysis. Energy-dispersive X-ray (EDX) analysis of the membranes was also performed in some cases with the same scanning electron microscope, by using an integrated Röntec QX2 EDS detector.

#### 2.3.3. Photocatalytic Activity

In addition, the photocatalytic activities of the TiO_2_-modified membranes (Area = 0.0017 m^2^) were determined by the photocatalytic decolorization of 50 mL of a methyl orange (MO) solution (10^−5^ M = 3.27 mg L^−1^) in a reactor equipped with a UV lamp (Lightech; 10 W, λ_max_ = 360 nm). Prior to this experiment, the following dyes were tested to determine the most feasible one to investigate the photocatalytic activity: acid red 1 (Synthesia, Pardubice, Czech Republic), methylene blue (Molar Chemicals Kft., Halásztelek, Hungary), and methyl orange (Sigma-Aldrich, Chemie GmbH, Saint Louis, MI, USA). Methyl orange was preferred due to its higher stability under UV light and lower adsorption to the PVDF membrane, and it is one of the most widely used model compounds in the field of photocatalysis [35,62,63].

First, the dye solution was kept in the dark for 15 h in the membrane-containing reactor to reach steady-state adsorption. After stabilization, the UV lamp was turned on (time 0) and samples were taken at regular intervals (time t) during the 30-h-long experiments. The absorbances were recorded with a spectrophotometer (Biochrom Biowave II+, Cambridge, UK).

The experiments were carried out at two different pH values since it is known that the photocatalytic activity of TiO_2_ varies with pH. The values chosen were either the natural pH of the methyl orange solution (pH ~ 5.3–5.6), which means no additional expenses in the process, or it was set to acidic condition (pH = 3), in which beneficial charges and interactions of TiO_2_ and methyl orange are known [62,63,64]. The absorbance was measured either at λ = 466 nm for the natural Ph and λ = 509 nm for the acidic condition. The Ph values were adjusted using a HCl solution (1 M).

To investigate the photocatalytic efficiency, the decolorization rate (ϵ) was calculated by the Equation (4):

(4) $$\epsilon =\frac{{\mathrm{Abs}}_{0}-{\mathrm{Abs}}_{t}}{{\mathrm{Abs}}_{0}}\times 100$$

where Abs_0_ and Abs_t_ are the measured absorbances of the dye solution when the lamp was turned on (time 0) and after a certain time of UV light irradiation (time t), respectively.

The decolorization of methyl orange with a low initial concentration follows pseudo-first-order kinetics; therefore, it can be described by the Langmuir-Hinshelwood kinetics law [21,29,35,62,65], using Equation (5):

(5) $$\ln\left(\frac{{\mathrm{Abs}}_{t}}{{\mathrm{Abs}}_{0}}\right)=-\;k\;t$$

where Abs_0_ and Abs_t_ are the measured absorbances of the dye after the steady-state was reached (time 0) and after a certain time of UV light irradiation (time t), respectively; k is the apparent first-order kinetics rate constant, expressed by the slope of the graph (h^−1^), and t is the reaction time (h). The initial reaction rate (r) was calculated from the initial concentration (C_0_):

(6) $$r=\;k\times {\;C}_{0}$$

The investigated responses for the statistical analysis were the pore size and reaction ra (r) of methyl orange decolorization under both natural and acidic conditions. The statistical analysis was performed in Statistica^TM^ Software (version 14.0.0.15, TIBCO Software Inc, Palo Alto, CA, USA).

## 3. Results and Discussion

### 3.1. Characterization of Neat Membranes

The fabricated conditions of neat membranes following the CCD and their characterization results are presented in Table 3.

The results of the ANOVA for the two responses (pure water flux and pore size) are presented in Table 4 and Table 5. At a 95% confidence level, the calculated probability is significant when its value is smaller than 0.05.

In a CCD, variables are investigated in three levels, and replicates of the central point are used to estimate the experimental errors. Furthermore, experiments are conducted in a random order, which minimizes experimental errors. Full factorial designs are alternative approaches to investigate the effect of factors, but the number of experiments is much higher than those studied in CCD (3^4^ = 81 experiments). Similarly, replicating all the experimental points significantly increases the time and costs of the study, which is not desirable [66,67]. Since the replicates in our experiments showed low standard deviation values, the developed synthesis protocol was reproducible, and, therefore, the standard deviation of the central point represents the deviations of all experimental points.

According to the presented ANOVA and the Pareto chart (Figure S1), the bath temperature and evaporation time did not cause significant effects in the studied intervals. At the same time, the amount of added PVDF and PVP significantly affected both the pure water flux and the pore size. The interaction between PVDF and PVP was significant for the pure water flux response, while the pore size was proportional to the square of PVP content.

Using these results and based on the principle of Occam’s razor, it is possible to exclude the non-significant factors and define the membrane behavior within the studied limits. This can be predicted from the regression analysis, and the equations can be written as follows:

(7) PWF=1268.7−47.1× PVDF+1671.3× PVP−82.2 x PVDF × PVP

(8) $$\mathrm{Pore}\;\mathrm{size}=0.32-0.016\times \;\mathrm{PVDF}+0.087\times \;\mathrm{PVP}-0.011\times {\;\mathrm{PVP}}^{2}$$

where PWF is the pure water flux (L m^−2^ h^−1^), pore size is expressed in µm, and PVDF and PVP are the amounts of the polymers used (wt%). The positive sign in front of the terms indicates that the response increases when the factor increases, while the negative sign indicates that the response reduces with increasing factor value. Excluding the non-significant factors, the predicted determination coefficients (R^2^_PWF_ = 0.68026, and R^2^_pore size_ = 0.79794) show that the observed and predicted values have a high correlation that can also be seen in Figure S2. It can be ascertained that the regression model is valid and provides a good explanation regarding the relationship between the variables and the response.

The pore size and water flux were not significantly affected by the bath temperature and evaporation time in the investigated levels. This does not mean that these parameters are not important. Much higher temperatures (e.g., 60 °C) can increase the solvent/non-solvent demixing rates in the bath, so the concentration of polymer on the surface increases, forming a dense skin layer with small pores on the membrane surface, while macrovoids can be formed in the structure of the membrane [32,33,68].

The investigated 1-min interval of evaporation time did not significantly affect the pure water flux and pore size. However, based on the literature, longer evaporation times can cause a significant change in the pore size, and there are complex processes behind the resulting effects. In a limited exposure time, non-solvent-induced phase separation (NIPS) is more predominant, whereby the majority of demixing happens in the coagulation bath. Within short time intervals, longer evaporation times result in the evaporation of more solvent, forming a skin layer with more polymer on the surface, creating a barrier that, upon immersion in the coagulation bath, would slow down the solvent/non-solvent demixing rate. This could result in membranes with more closed substructure, smaller pores, lower permeance, and higher rejections [4,48,69,70]. The opposite can happen if much longer exposure times are used: vapor-induced phase separation (VIPS) takes place predominantly, and demixing happens mainly between the solvent and air humidity, which completely modifies the morphology of membranes. Specifically, much longer air exposure results in the absorption of more water from the air humidity in the surface of the membrane. This happens slowly and homogeneously, hence symmetric membranes with internal and surface pores can be formed [21,32,48,71].

**Figure 1:**
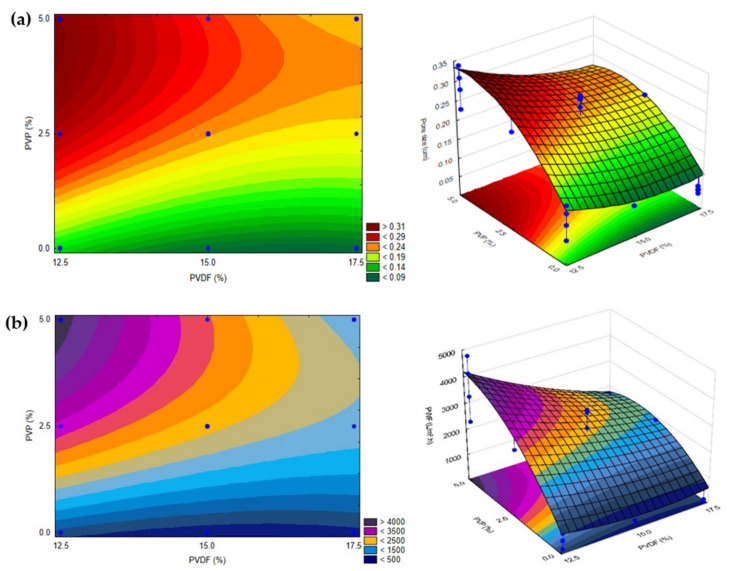
2 and 3-dimensional surfaces showing the dependence of (**a**) pore size and (**b**) pure water flux on the concentrations of PVP and PVDF at a fixed bath temperature of 20 °C and 30 s of evaporation time. Blue dots represent experimental values within the investigated design space (*n* = 27).

Fitted surfaces were generated to represent the effects of the factors (bath temperature, evaporation time, PVDF and PVP content) on the responses (pure water flux and pore size). Since the bath temperature and evaporation time did not affect significantly the responses within the studied intervals, it is possible to represent a fitted surface for pure water flux and pore size as functions of PVDF and PVP concentrations, considering their center values (20 °C bath temperature and 30 s evaporation time; Figure 1). The model shows that both the pure water flux and pore size increase when the concentration of PVP increases and the concentration of PVDF decreases, as expected. PVP is known to be a pore former and its presence increases the thermodynamic instability of the casting solution. This leads to instantaneous demixing in the coagulation bath and thereby higher PVP concentration, which facilitates the formation of macrovoids in the structure of the membrane [72,73]. The opposite happens while increasing the concentration of PVDF: the precipitation process slows down, resulting in smaller pore size and therefore lower pure water flux [36].

It is important to investigate the contents of the additive and polymer simultaneously, since greater total polymer and additive concentrations affect the viscosity of the casting solution. Therefore, this also affects the casting performance and the characteristics of the formed membranes. If the viscosity of a casting solution is higher, then the demixing exchange in the bath will be delayed and the formation of macrovoids can be suppressed [53,74]. This is disadvantageous if the goal is to fabricate membranes with large pores. This deduction is reinforced by the results in Figure 1, representing that higher PVDF and PVP contents resulted in membranes with lower PWF and pore size.

Using polymers either in lower or higher concentrations beyond the studied intervals might result in membranes with undesired characteristics: a too high concentration of PVDF and too low concentration of PVP can lead to membranes with no permeability and very low flux. Nevertheless, using PVP in too high and PVDF in too low concentrations can result in inadequate membranes with holes instead of pores and no retention capability. Using PVP and PVDF both at higher concentrations can increase the viscosity of the solution considerably. This results in sponge-like membranes with no pores, or a heterogeneous casting solution that yields membranes with holes.

Based on Figure 1, the pore size and pure water flux would increase while the concentration of PVP increases and the concentration of PVDF decreases, and the same would happen under the opposite circumstances. Hence, for the fabrication of membranes with the desired properties, it is necessary to take into consideration the type of wastewater to be purified. Consequently, the chosen fabrication conditions depend on the required molecular weight cut-off value, the average size of molecules/particles, the necessary flux, and the required rejection efficiency. The fitted surface presented in Figure 1 is suitable to be used as a basis to fabricate membranes with the desired properties, which is demonstrated with some examples as follows: (1) for protein elimination ultrafiltration is necessary, thus the bottom part of the fitted surface should be chosen (e.g., 12.5% PVDF and 0% PVP, or 15% PVDF and 0–1.0% PVP); (2) microfiltration with 0.2–0.3 µm pore membranes can be achieved using intermediate values for the variables (e.g., 14.0–16.0% PVDF and 2–3% PVP).

### 3.2. Characterization of TiO_2_-Modified Membranes

#### 3.2.1. Filtration and Morphology

During the fabrication of the neat membrane, applying the minimum amount of PVDF (12.5%) combined with the maximum amount of PVP additive (5%) resulted in weak membranes with too many macrovoids. Hence, to be able to add up to 3% of TiO_2_ and fabricate well-formed, homogeneous casting solutions with reasonable viscosity, the maximum value of PVP was set to 3% to ensure that the obtained membranes contained a maximum of 6% of total additives.

The fabrication conditions used for the preparation of TiO_2_-modified membranes following the CCD and the results of their characterization are presented in Table 6. The evaporation time and bath temperature were fixed at 30 s and 20 °C, respectively, based on the previously discussed results.

The results of the ANOVA for the pore size response are presented in Table 7.

Based on Table 7, only PVP varied the membrane pore size significantly within the studied intervals, and this variation is linear. The regression analysis can be predicted by excluding the non-significant variables:

(9) Pore size=0.1205+0.05494 × PVP

where the pore size is expressed in µm, and PVP is the concentration of the additive (wt%). The predicted determination coefficient excluding the non-significant factors is R^2^ = 0.70426, which means that the observed and predicted values have a high correlation, which can also be seen in Figure S3. Accordingly, it can be ascertained that the regression model is valid and provides a good prediction.

**Figure 2:**
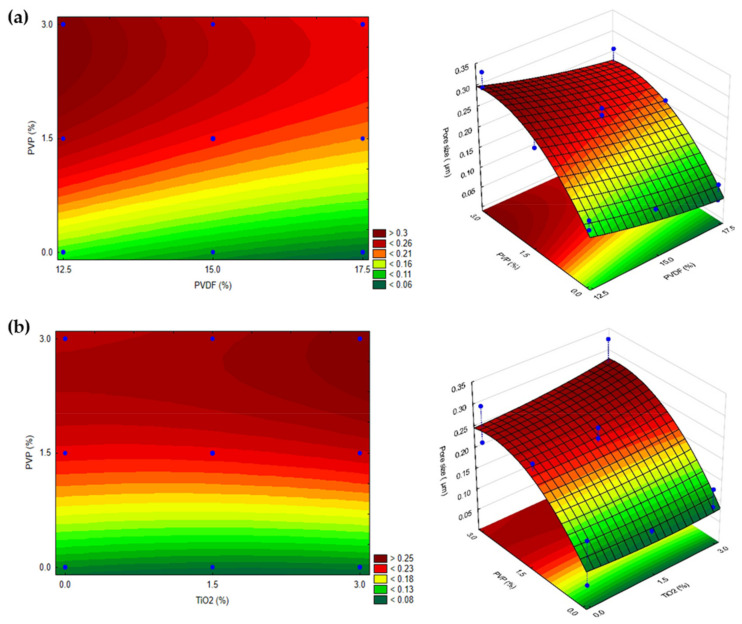
2 and 3-dimensional surfaces representing the correlation between pore size and PVP concentration in TiO_2_-modified membranes using different amounts of (**a**) PVDF and (**b**) TiO_2_. Blue dots represent experimental values within the investigated design space (*n* = 17).

Since only the addition of PVP (up to 3 wt%) had a significant effect, varying the concentrations of TiO_2_ and PVDF did not alter the response significantly. This can be confirmed based on Figure 2, in which the correlation between the pore size and the concentrations of PVDF and TiO_2_ was nearly parallel to their axes.

The neat and modified membranes showed similar changes in the pore size responses when different amounts of PVP and PVDF were used due to the previously discussed reasons. This can also be confirmed by comparing Figure 1b with Figure 2a.

**Figure 3:**
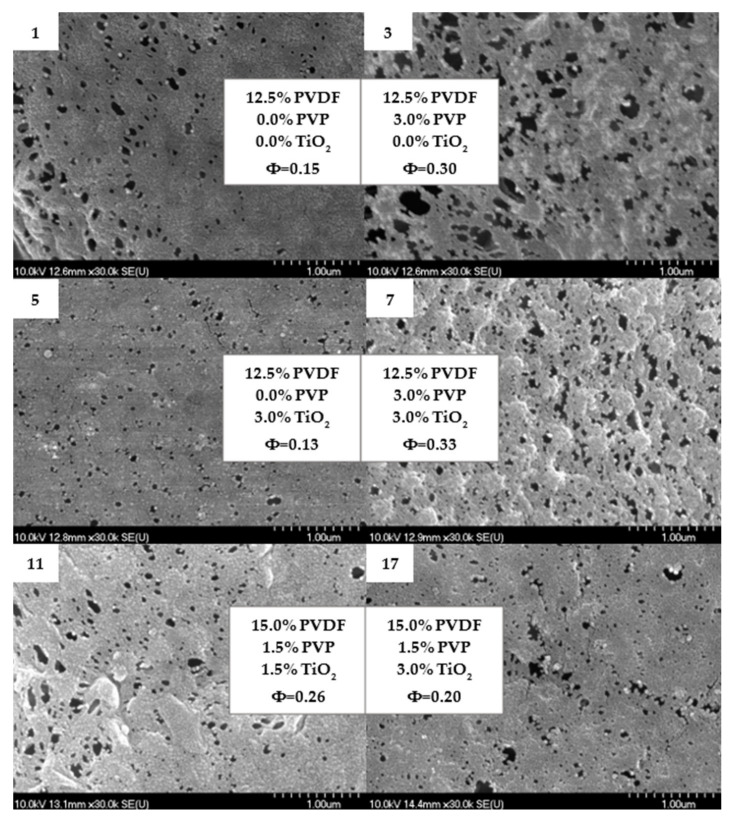
Scanning electron microscopy images of the top surface of 6 different membranes containing PVDF, PVP and TiO_2_. Φ is the calculated average pore size in µm.

**Figure 4:**
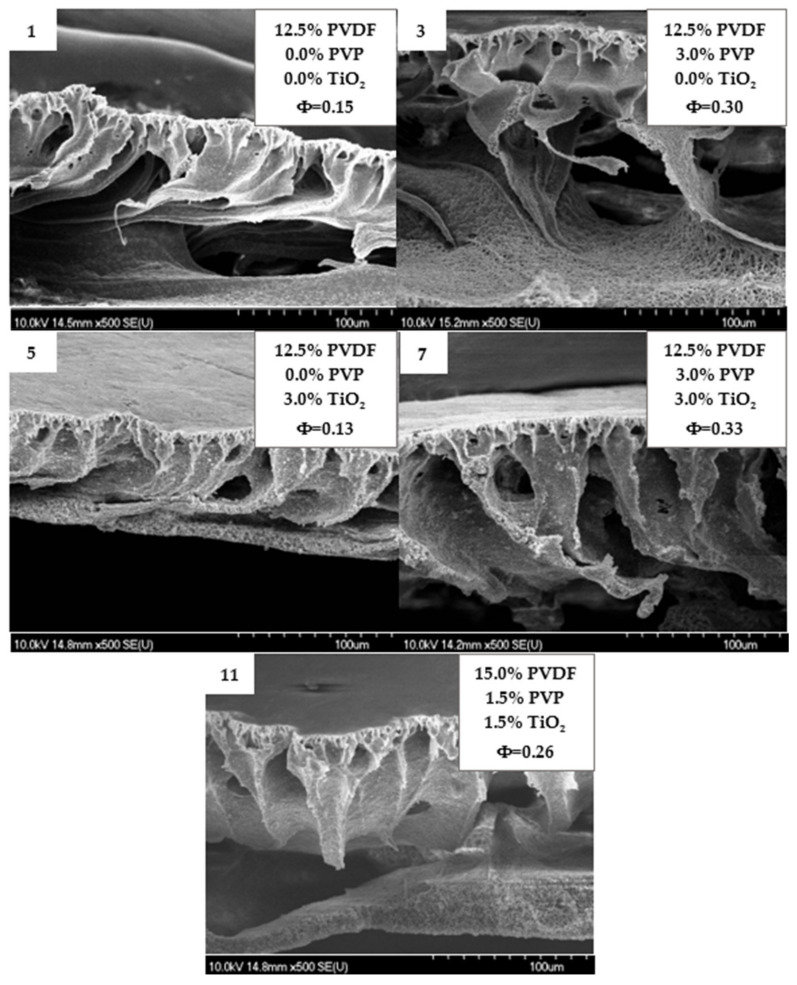
Scanning electron microscopy images of the cross-section of five different membranes containing PVDF, PVP and TiO_2_. Φ is the calculated average pore size in µm.

The addition of TiO_2_ did not significantly change the pore size of the membranes. It is widely known that hydrophilic nanoparticles can enhance pure water flux compared to that for neat PVDF membranes [26,54,75]. However, hydrophilic polymer additives (such as PVP) have a greater influence on pore size and the enhancement of pure water flux due to their “pore former” characteristic [30,72]. It is worth noting the complexity of the phase-inversion method that can be affected by thermodynamic and kinetic effects. The addition of hydrophilic PVP can induce demixing between the solvent and non-solvent. It can also enhance the viscosity of the casting solution and delay phase separation, thus having the opposite effect on the characteristics of membranes [30,73]. The discussed pore enhancement can be confirmed based on the SEM images presented in Figure 3, which is in good agreement with the literature [21,73]. From Figure 3, areas containing agglomerated nanoparticles can be observed; however, for the most part, TiO_2_ is uniformly dispersed on the surface of the membrane and entrapped in some holes (Figure 4). The homogeneous dispersion of TiO_2_ nanoparticles both on the surface and cross-section was also proven by SEM images obtained during EDX elemental mapping (Figures S4 and S5).

Figure 4 shows the cross-section of some membranes, which present asymmetric layers with irregular macrovoids that can be explained by the usage of NMP as solvent. This resulted in a fast phase-inversion process due to the huge affinity of NMP to water, as expected [76,77]. Due to the fast phase-inversion rate, a skin layer was formed at the very beginning of the demixing process which was responsible for limiting further exchange, resulting in irregular macrovoids beneath this layer. Moreover, the hydrophobicity of PVDF also facilitated the fast formation of the skin layer due to repulsive forces between the polymer and water [78].

#### 3.2.2. Photocatalytic Activity

**Figure 5:**
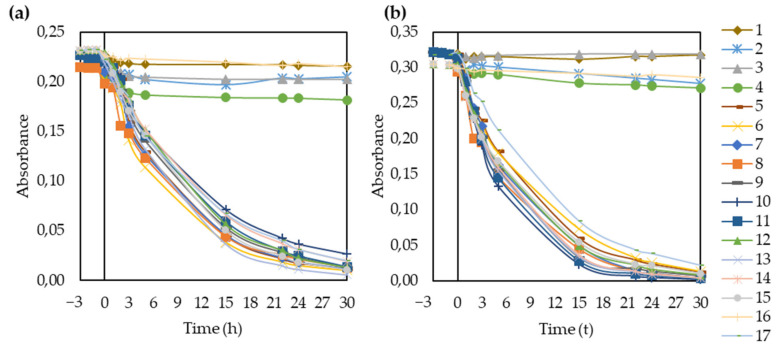
Absorbance of methyl orange for all fabricated membranes during adsorption and UV irradiation, under both (**a**) natural and (**b**) acidic (pH = 3) conditions.

**Figure 6:**
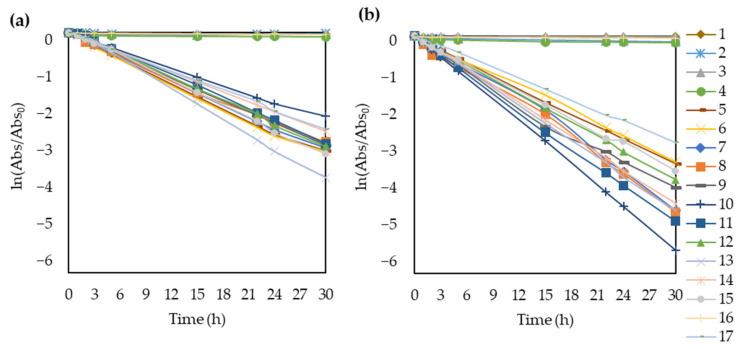
Apparent rate constant calculation plotting the ln(Abs/Abs_0_) of methyl orange as a function of time under (**a**) natural pH and (**b**) acidic (pH = 3) conditions.

The photocatalytic activities were calculated from the decolorization efficiency of the TiO_2_-modified membranes for methyl orange under both natural and acidic conditions. Over the 30 h of UV irradiation, the concentration decreased exponentially, as expected (Table S1 and Figure 5). Hence, it was possible to use the Langmuir-Hinshelwood model to plot ln (Abs/Abs_0_) as a function of time and calculate the apparent rate constants (k) based on the slopes of the lines (Figure 6).

The membranes without TiO_2_ (1, 2, 3, 4, and 16) had negligible photocatalytic activities for methyl orange. After 30 h, all the TiO_2_-containing membranes almost completely decomposed the dye under both pH conditions. The porous structure is beneficial to photocatalysis because the pores can serve as a pathway for the dye to reach the interior surface of membranes. The dye was adsorbed both on the TiO_2_ particles fixed in the pores of the membrane and on the polymer surface. In the latter case, the dye molecules were still close enough to the TiO_2_ particles to undergo photocatalytic degradation [21,35]. The rate of decolorization decreased after 15 h because it was less and less likely for the dye molecules to collide with hydroxyl radicals formed by the TiO_2_ nanoparticles since most of the organic molecules were already oxidized [79].

In addition, the point of zero charge of TiO_2_ is at pH 6.8, which means that its surface is positively charged at pH values lower than that [64]. Therefore, the photocatalyst had a positively charged surface in the studied pH range (3–5.6). Meanwhile, the methyl orange was negatively charged in the same pH range. Therefore, reducing the pH from 5.6 to 3 resulted in higher attraction forces between the surface of TiO_2_ and the dye molecules, enhancing the photocatalytic activity. This explains the good dye removal efficiency within the studied pH range and the more efficient decolorization at pH = 3 for all TiO_2_-modified membranes. This is in good agreement with the literature [35,62,63,79]. Based on the absorbance spectra showing the decomposition of the dye (Figure S6), the MO solution lost its color due to photocatalytic degradation, while the produced oxidation by-products increased the absorbance in the 200–240 nm region.

The results of the photocatalytic activity measurements are summarized in Table 8.

It is worth highlighting that the membranes without TiO_2_ (membranes 1, 2, 3, 4, and 16), did not decompose methyl orange. Therefore, both the calculated apparent rate constants (k) and reaction rates (r) are negligible for these membranes just like the slopes of the ln (Abs/Abs_0_) fitted curves. Moreover, the Langmuir-Hinshelwood model does not explain the kinetics in these cases and the determination coefficients are also far from 1.0.

For the TiO_2_-modified membranes, it was possible to evaluate how the different fabrication conditions affect their photocatalytic activity. For this purpose, their rate constants (r) were used as a response for the statistical analysis under both natural and acidic conditions. The ANOVA results are presented in Table 9 and Table 10.

According to the presented ANOVA and the Pareto chart (Figure S7), the concentration of TiO_2_ significantly affected the achievable photocatalytic efficiency both under natural and acidic conditions. The variations were linear and quadratic, as expected according to the discussion already presented.

Using Occam’s razor once again, the non-significant factors can be excluded, and the predicted regression analysis can be modeled as follows:

(10) $$r_{\mathrm{natural}}=0.310550\;\times {\;\mathrm{TiO}}_{2}-0.068526\;\times {\;\mathrm{TiO}}_{2}{}^{2}$$

(11) $$r_{\mathrm{acid}}=0.487122\;\times {\;\mathrm{TiO}}_{2}-0.119827\;\times {\;\mathrm{TiO}}_{2}{}^{2}$$

where r is the reaction rate of methyl orange decolorization (mg L^−1^ h^−1^) and TiO_2_ is the concentration of the photocatalyst (wt%). The predicted determination coefficients were 0.94401 and 0.9178 for natural and acidic conditions, respectively. These values are in remarkable agreement with the observed values, which can also be seen in Figure S8.

**Figure 7:**
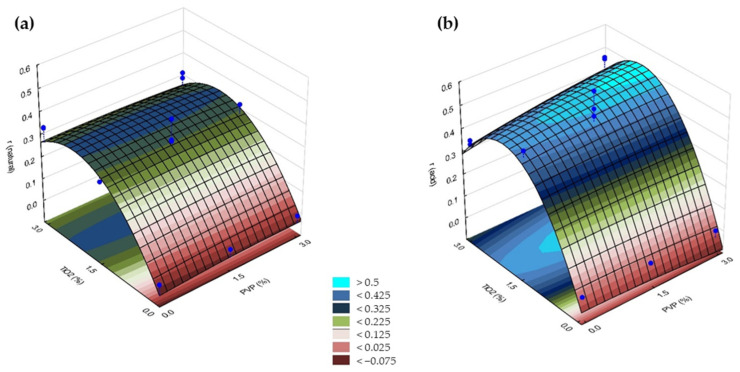
Fitted surfaces of reaction rate (r) responses obtained by varying the concentration of PVP for the membranes containing TiO_2_ at different concentrations under (**a**) natural and (**b**) acidic conditions. Blue dots represent experimental values within the investigated design space (*n* = 17).

As mentioned above, both the concentrations of PVDF and PVP had no significant effect on the photocatalytic efficiency within the studied intervals. A parallel response surface was obtained as the concentrations of PVDF and PVP varied, which means that the response changes only when varying the concentration of the photocatalyst (Figure 7).

Based on Figure 7, it can be seen that using TiO_2_ in the higher concentration (3.0%) did not increase the decolorization rate of MO. This can be possibly explained by the agglomeration of photocatalyst nanoparticles in the polymer matrix, which can reduce the surface area and light absorption ability, as was also described by Hir et al. [35]. This shows that using TiO_2_ at 1.5% could be the optimum concentration for the conditions investigated in this study.

In general, TiO_2_ particles are entrapped both in the top surface and the deeper part of the membrane; therefore, photocatalysis takes place in both places. The TiO_2_ present in the surface of the membrane can rapidly decolorize the dye solution. However, those nanoparticles immobilized on the surfaces of pores and hollows also contribute to the degradation of organic contaminants, which makes the photocatalytic decomposition of organic pore-foulants possible. The TiO_2_ present in the inner layers affects the penetration of UV light into the membrane, and therefore, the decolorization of the dye. This is because in order for photocatalysis to take place, UV light must be able to penetrate into the membrane material and interact with TiO_2_ [80,81]. Thus, adding more TiO_2_ does not necessarily increase the photocatalytic activity of the membrane. This explains why using TiO_2_ at only 1.5% could be more beneficial for the conditions investigated in this study.

In relation to this statement, additional measurements were carried out with a UV radiometer (Optix Tech Inc., UVTEX a+b idm, District of Columbia, WA, USA). It was ascertained that the neat membrane showed 6.3% transmittance to the UV irradiation applied. Meanwhile, TiO_2_-containing membranes had 0.0% transmittance, even in the case of lower TiO_2_ contents. This indirectly proves that nanoparticle content considerably affects the penetration depth of UV photons.

In addition, Sun et al. [28] found that PVDF combined with TiO_2_ can enhance the decolorization of the dye to some extent. This was not only because of the presence of the photocatalyst, but also due to the capability of the polymer to enhance the transport of photogenerated electrons and holes. As a result, the recombination of charge carriers was suppressed, and the photocatalytic activity of the membrane was improved.

In summary, due to the complex effects discussed above, adding 1.5% of TiO_2_ to the dope solution was enough to achieve good photocatalytic performance for the fabricated membranes. At the same time, the contents of PVP and PVDF can be chosen according to the requirements for the membranes, based on the presented fitted surface in Figure 2.

## 4. Conclusions

This research aimed to combine different studies that investigated the effects of various membrane fabrication conditions on membrane properties and performances to help researchers in developing PVDF membranes with desired properties. Membranes were fabricated by the phase-inversion method using PVDF and PVP dissolved in NMP, and TiO_2_ nanoparticles were incorporated into the membrane matrices, and the effects of experimental conditions were analyzed by CCD statistical analysis.

Initially, neat membranes were fabricated, and the effects of four different variables -bath temperature, evaporation time, PVDF and PVP contents—were analyzed. Bath temperature and evaporation time did not significantly affect water flux and the structure of the membranes within the studied intervals (15–25 °C and 0–60 s). Using any value within these intervals resulted in membranes of very similar qualities. The polymer (12.5–17.5% PVDF) and the pore former additive (0–5% PVP) significantly altered the pore size and pure water flux of the membranes. Using more PVDF increased the viscosity of the dope solution, which affected the precipitation process by reducing the demixing rate, resulting in smaller pore size and lower pure water flux. The opposite happened when more PVP was added: instantaneous demixing resulted in bigger pore size and higher pure water flux, which should only be used to a limited extent to avoid possible hole formation. Using a dope solution of inappropriate composition can result in membranes either without pores or with macrovoids, reducing their performance and rejection rate. Three-dimensional response surfaces and equations were generated based on which it is possible to predict and define the best conditions for the fabrication of membranes with specific attributes.

In the case of photocatalytic membranes, three variables were investigated: PVDF, PVP, and TiO_2_ contents. Within the studied interval, the addition of TiO_2_ (0–3%) did not significantly affect pore size or pure water flux (these parameters were rather dependent on the concentration of PVP). However, the incorporated TiO_2_ nanoparticles endowed the prepared membranes with remarkable photocatalytic properties. The addition of 1.5% of TiO_2_ resulted in the rapid decolorization of methyl orange under both natural and acidic conditions; however, in the latter case, the rates were higher due to the anionic characteristic of the dye and the beneficial surface charge of the TiO_2_ that enhanced the adsorption of the dye on the nanoparticle. As a result, additional three-dimensional response surfaces and equations were generated to predict the pore size and the decolorization rate of the photocatalytic membranes. These models can be used to guide further research.

The investigation of how different variables affect the characteristics and performance of photocatalytic membranes fabricated via phase-inversion is an important step towards their potential industrial application. This study shows how certain fabrication conditions affect their performance and, using the modeled results, it is possible to set conditions that are the most suitable to prepare membranes for various purposes.

## Figures and Tables

**Table 1:** Independent variable values for the central composite design of the neat membrane.

Variable	Level		
	−1	0	+1
Bath temperature (°C)	15	20	25
Evaporation time (s)	0	30	60
PVDF (wt%)	12.5	15.0	17.5
PVP (wt%)	0	2.5	5.0

**Table 2:** Independent variable values for the central composite design of TiO_2_-modified membranes.

Variable	Level		
	−1	0	+1
PVDF (wt%)	12.5	15.0	17.5
PVP (wt%)	0	1.5	3.0
TiO_2_ (wt%)	0	1.5	3.0

**Table 3:** Fabrication conditions according to the central composite design and characterization results of neat membranes (27 experiments: 2^4^ = 16 + 3 (C) center points + 8 (S) star-points).

Membrane	Variable Factors				Membrane Characterization		
	Bath T	Evap. Time	PVDF	PVP	Porosity	Pure Water Flux	Pore Size
	(°C)	(s)	(%)	(%)	(%)	(L m^−2^ h^−1^)	(µm)
1	15	0	12.5	0.0	89.2	715	0.13
2	25	0	12.5	0.0	82.2	107	0.06
3	15	60	12.5	0.0	92.2	420	0.10
4	25	60	12.5	0.0	89.0	461	0.15
5	15	0	17.5	0.0	83.4	54	0.04
6	25	0	17.5	0.0	85.6	130	0.06
7	15	60	17.5	0.0	89.0	119	0.05
8	25	60	17.5	0.0	85.8	110	0.05
9	15	0	12.5	5.0	89.8	2309	0.23
10	25	0	12.5	5.0	91.4	4150	0.31
11	15	60	12.5	5.0	89.7	4801	0.34
12	25	60	12.5	5.0	90.3	3277	0.28
13	15	0	17.5	5.0	88.1	1102	0.16
14	25	0	17.5	5.0	87.3	1777	0.21
15	15	60	17.5	5.0	88.0	1063	0.16
16	25	60	17.5	5.0	87.0	1088	0.17
17 (C)	20	30	15.0	2.5	88.6	2482	0.25
18 (C)	20	30	15.0	2.5	88.5	3186	0.28
19 (C)	20	30	15.0	2.5	89.1	3156	0.27
20 (S)	15	30	15.0	2.5	89.0	1378	0.18
21 (S)	25	30	15.0	2.5	94.5	2106	0.21
22 (S)	20	0	15.0	2.5	89.3	3070	0.27
23 (S)	20	60	15.0	2.5	89.7	480	0.11
24 (S)	20	30	12.5	2.5	87.1	2517	0.25
25 (S)	20	30	17.5	2.5	87.0	1949	0.22
26 (S)	20	30	15.0	0.0	86.5	241	0.08
27 (S)	20	30	15.0	5.0	89.0	2533	0.25

**Table 4:** Analysis of variance of pure water flux (L m^−2^ h^−1^). R^2^ = 0.8243; Pure error = 158,465.3. (L) is the linear relation, (Q) is the quadratic relation of the factors, and Df is the degrees of freedom of the analysis.

Factor	Sum of Squares	Df	Mean Square	F-Value	Prob > F
1. Bath (°C) (1 L)	86,070	1	86,070	0.5431	0.537861
Bath (°C) (1 Q)	228,501	1	228,501	1.4420	0.352747
2. Ev. Time (s) (2 L)	141,465	1	141,465	0.8927	0.444475
Ev. time (s) (2 Q)	180,617	1	180,617	1.1398	0.397493
3. PVDF (%) (3 L)	7,175,580	1	7,175,580	45.2817	0.021378 ^a^
PVDF (%) (3 Q)	95,960	1	95,960	0.6056	0.517910
4. PVP (%) (4 L)	21,655,116	1	21,655,116	136.6552	0.007238 ^a^
PVP (%) (4 Q)	1,096,213	1	1,096,213	6.9177	0.119247
1 L by 2 L	744,338	1	744,338	4.6972	0.162524
1 L by 3 L	64,643	1	64,643	0.4079	0.588403
1 L by 4 L	143,831	1	143,831	0.9076	0.441288
2 L by 3 L	348,395	1	348,395	2.1986	0.276366
2 L by 4 L	38,711	1	38,711	0.2443	0.670080
3 L by 4 L	4,219,943	1	4,219,943	26.6301	0.035561 ^a^
Lack of Fit	8,219,698	10	821,970	5.1871	0.172349
Pure Error	316,931	2	158,465		
Total SS	48,585,198	26			

^a^ Statistically significant (*p* < 0.05). Standard deviation = 398.08.

**Table 5:** Analysis of variance of pore size (µm). R^2^ = 0.8514; Pure error = 0.0002333. (L) is the linear relation, (Q) is the quadratic relation of the factors, and Df is the degrees of freedom of the analysis.

Factor	Sum of Squares	Df	Mean Square	F-Value	Prob > F
1. Bath (°C) (1 L)	0.000653	1	0.000653	2.8004	0.236216
Bath (°C) (1 Q)	0.000962	1	0.000962	4.1234	0.179399
2. Ev. Time (s) (2 L)	0.000225	1	0.000225	0.9639	0.429724
Ev. time (s) (2 Q)	0.001873	1	0.001873	8.0255	0.105288
3. PVDF (%) (3 L)	0.029595	1	0.029595	126.8349	0.007792 ^a^
PVDF (%) (3 Q)	0.001150	1	0.001150	4.9303	0.156549
4. PVP (%) (4 L)	0.106963	1	0.106963	458.4112	0.002174 ^a^
PVP (%) (4 Q)	0.007183	1	0.007183	30.7827	0.030984 ^a^
1 L by 2 L	0.000400	1	0.000400	1.7143	0.320634
1 L by 3 L	0.000400	1	0.000400	1.7143	0.320634
1 L by 4 L	0.000400	1	0.000400	1.7143	0.320634
2 L by 3 L	0.002025	1	0.002025	8.6786	0.098496
2 L by 4 L	0.000025	1	0.000025	0.1071	0.774506
3 L by 4 L	0.003025	1	0.003025	12.9643	0.069222
Lack of Fit	0.030382	10	0.003038	13.0210	0.073383
Pure Error	0.000467	2	0.000233		
Total SS	0.207600	26			

^a^ Statistically significant (*p* < 0.05). Standard deviation = 0.0153.

**Table 6:** Fabrication conditions according to the central composite design and characterization results of the modified membranes (17 experiments: 2^3^ = eight experiments + three (C) center points + six (S) star-points).

Membrane	Variable Factors			Membrane Characterization		
	PVDF	PVP	TiO_2_	Porosity	Pure Water Flux	Pore Size
	(%)	(%)	(%)	(%)	(L m^−2^ h^−1^)	(µm)
1	12.5	0.0	0.0	87.8	921	0.15
2	17.5	0.0	0.0	80.6	73	0.05
3	12.5	3.0	0.0	89.7	3737	0.30
4	17.5	3.0	0.0	87.0	1791	0.21
5	12.5	0.0	3.0	84.4	609	0.13
6	17.5	0.0	3.0	80.8	247	0.08
7	12.5	3.0	3.0	88.5	4592	0.33
8	17.5	3.0	3.0	61.6	1474	0.26
9 (C)	15.0	1.5	1.5	86.9	1593	0.20
10 (C)	15.0	1.5	1.5	87.3	2692	0.26
11 (C)	15.0	1.5	1.5	87.1	2689	0.26
12 (S)	12.5	1.5	1.5	89.0	2310	0.23
13 (S)	17.5	1.5	1.5	84.9	1685	0.21
14 (S)	15.0	0.0	1.5	84.1	372	0.10
15 (S)	15.0	3.0	1.5	86.8	2092	0.23
16 (S)	15.0	1.5	0.0	87.2	2358	0.24
17 (S)	15.0	1.5	3.0	87.7	1651	0.20

**Table 7:** Analysis of variance of the pore size (µm). R^2^ = 0.92384; Pure error = 0.0011628. (L) is the linear relation, (Q) is the quadratic relation of the factors, and Df is the degrees of freedom of the analysis.

Factor	Sum of Squares	Df	Mean Square	F-Value	Prob > F
1. PVDF (%) (1 L)	0.010595	1	0.010595	9.11185	0.094455
PVDF (%) (1 Q)	0.000108	1	0.000108	0.09327	0.788915
2. PVP (%) (2 L)	0.067912	1	0.067912	58.40530	0.016694 ^a^
PVP (%) (2 Q)	0.007158	1	0.007158	6.15642	0.131211
3. TiO_2_ (%) (3 L)	0.000390	1	0.000390	0.33521	0.621125
TiO_2_ (%) (3 Q)	0.000085	1	0.000085	0.07345	0.811791
1 L by 2 L	0.000007	1	0.000007	0.00614	0.944700
1 L by 3 L	0.000710	1	0.000710	0.61103	0.516245
2 L by 3 L	0.000649	1	0.000649	0.55833	0.532840
Lack of Fit	0.005018	5	0.001004	0.86316	0.614004
Pure Error	0.002326	2	0.001163		
Total SS	0.096430	16			

^a^ Statistically significant (*p* < 0.05). Standard deviation = 0.0346.

**Table 8:** Decolorization of methyl orange (C_0_ = 10^−5^ M~3.27 mg L^−1^) at different pH ranges according to the central composite design and characterization results of the modified membranes (17 experiments: 2^3^ = eight experiments + three (C) center points + six (S) star-points). Apparent rate constants k (h^−1^) were calculated from the slope of ln(Abs/Abs_0_) versus time, R^2^ is the correlation coefficient of the generated curve, ϵ_15_ is the decolorization efficiency after 15 h of experiment (%), and r is the initial reaction rate (mg L^−1^ h^−1^).

Membrane	Natural pH (5.3~5.6)				Acid pH (3)			
	k	R^2^	ϵ_15_	r	k	R^2^	ϵ_15_	r
1	~0.0	0.0382	4	~0.0	~0.0	0.0057	3	~0.0
2	~0.0	0.4193	6	~0.0	~0.0	0.9093	6	~0.0
3	~0.0	0.6276	5	~0.0	~0.0	0.4191	0	~0.0
4	~0.0	0.7066	10	~0.0	~0.0	0.9439	7	~0.0
5	−0.1034	0.9998	80	0.3381	−0.1070	0.9997	81	0.3499
6	−0.1043	0.9951	81	0.3411	−0.1018	0.9984	75	0.3329
7	−0.0999	0.9976	78	0.3267	−0.1443	0.9969	84	0.4719
8	−0.0926	0.9975	78	0.3028	−0.1420	0.9962	85	0.4643
9 (C)	−0.0948	0.9998	76	0.3100	−0.1296	0.9946	90	0.4238
10 (C)	−0.0729	0.9967	67	0.2384	−0.1798	0.9999	93	0.5879
11 (C)	−0.0939	0.9992	73	0.3071	−0.1566	0.9997	91	0.5121
12 (S)	−0.0959	0.9991	75	0.3136	−0.1204	0.9998	84	0.3937
13 (S)	−0.1240	0.9991	83	0.4055	−0.1471	0.9997	89	0.4810
14 (S)	−0.0825	0.9999	71	0.2698	−0.1413	0.9997	88	0.4621
15 (S)	−0.1031	0.9991	78	0.3371	−0.1122	0.9992	82	0.3669
16 (S)	~0.0	0.9106	3	~0.0	~0.0	0.9104	2	~0.0
17 (S)	−0.0813	0.9990	70	0.2659	−0.0904	0.9996	74	0.2956

**Table 9:** Analysis of variance of initial reaction rates (r) at the natural pH of methyl orange. R^2^ = 0.9659; Pure error = 0.0016424. (L) is the linear relation, (Q) is the quadratic relation of the factors, and Df is the degrees of freedom of the analysis.

Factor	Sum of Squares	Df	Mean Square	F-Value	Prob > F
1. PVDF (%) (1 L)	0.000504	1	0.000504	0.3069	0.635249
PVDF (%) (1 Q)	0.006677	1	0.006677	4.0653	0.181309
2. PVP (%) (2 L)	0.000031	1	0.000031	0.0189	0.903347
PVP (%) (2 Q)	0.000102	1	0.000102	0.0623	0.826221
3. TiO_2_ (%) (3 L)	0.247937	1	0.247937	150.9559	0.006559 ^a^
TiO_2_ (%) (3 Q)	0.083634	1	0.083634	50.9203	0.019078 ^a^
1 L by 2 L	0.000090	1	0.000090	0.0551	0.836300
1 L by 3 L	0.000055	1	0.000055	0.0332	0.872132
2 L by 3 L	0.000309	1	0.000309	0.1880	0.706881
Lack of Fit	0.009209	5	0.001842	1.1213	0.533581
Pure Error	0.003285	2	0.001642		
Total SS	0.366334	16			

^a^ Statistically significant (*p* < 0.05). Standard deviation = 0.0405.

**Table 10:** Analysis of variance of initial reaction rates (r) determined under acidic conditions (pH = 3) in methyl orange solution. R^2^ = 0.93389; Pure error = 0.0067452. (L) is the linear relation, (Q) is the quadratic relation of the factors, and Df is the degrees of freedom of the analysis.

Factor	Sum of Squares	Df	Mean Square	F-Value	Prob > F
1. PVDF (%) (1 L)	0.000393	1	0.000393	0.05828	0.831726
PVDF (%) (1 Q)	0.000654	1	0.000654	0.09693	0.784996
2. PVP (%) (2 L)	0.002503	1	0.002503	0.37104	0.604416
PVP (%) (2 Q)	0.000140	1	0.000140	0.02075	0.898660
3. TiO_2_ (%) (3 L)	0.366569	1	0.366569	54.34502	0.017908 ^a^
TiO_2_ (%) (3 Q)	0.201042	1	0.201042	29.80503	0.031952 ^a^
1 L by 2 L	0.000011	1	0.000011	0.00164	0.971398
1 L by 3 L	0.000076	1	0.000076	0.01121	0.925327
2 L by 3 L	0.008026	1	0.008026	1.18995	0.389238
Lack of Fit	0.034477	5	0.006895	1.02226	0.562020
Pure Error	0.013490	2	0.006745		
Total SS	0.725524	16			

^a^ Statistically significant (*p* < 0.05). Standard deviation = 0.0821.

## Supplementary Materials

The following are available online at https://www.mdpi.com/article/10.3390/polym14010113/s1, Figure S1. Pareto chart for the pure water flux (a) and pore size (b) of the neat-fabricated membranes. Variables with *p* > 0.05 are considered significant; Figure S2. Predicted x observed values for the regression analysis of pure water flux (a) and pore size (b) of the neat-fabricated membranes; Figure S3. Pareto chart (a) and predicted x observed values (b) for the regression analysis of the pore size response of TiO_2_-modified membranes; Figure S4. SEM and EDX mapping images for membranes 11 (1.5% TiO_2_) (a) and 17 (3.0% TiO_2_) (b), from the top surface (a_1_, b_1_) and the cross sections (a_2_, b_2_); Figure S5. EDX spectra representing the elemental composition of membranes 11 (1.5% TiO_2_) (a) and 17 (3.0% TiO_2_) (b), from the top surface (a_1_, b_1_) and the cross sections (a_2_, b_2_); Figure S6. Changes in the absorption spectra of the methyl orange solution during photocatalytic decomposition with TiO_2_. A0 is the spectrum in the beginning of the experiment (*t* = 0) and At is the spectrum in each different time interval (t); Figure S7. Pareto chart for the r response of the natural (a) and acid conditions (b) of the TiO_2_-modified membranes. Variables with *p* > 0.05 are considered significant; Figure S8. Predicted x observed values of r response for the regression analysis of natural (a) and acidic conditions (b) of the TiO_2_ modified fabricated membranes; Table S1. Absorbance values during the photocatalytic experiments using methyl orange in natural pH conditions (λ = 509 nm) and acidic conditions (λ = 466 nm) for the adsorption time and 30 h UV irradiation.
