# Supplementary material for: Statistical Analysis of Synthesis Parameters to Fabricate PVDF/PVP/TiO2 Membranes via Phase-Inversion with Enhanced Filtration Performance and Photocatalytic Properties
Source: Polymers (Basel). 2021 Dec 29;14(1):113. doi: 10.3390/polym14010113 (PMC8747740; doi:10.3390/polym14010113)
Supplement: Supplementary file 1 [file polymers-14-00113-s001.zip › polymers-1443711-supplementary.pdf]

# Statistical analysis of synthesis parameters to fabricate PVDF/PVP/$TiO_2$ membranes via phase-inversion with enhanced filtration performance and photocatalytic properties

**Erika Nascimben Santos [1,2], Ákos Fazekas [1,2], Cecilia Hodúr [1], Zsuzsanna László [1], Sándor Beszédes [1], Daniele Scheres Firak [2,3], Tamás Gyulavári [4], Klára Hernádi [4,5], Gangasalam Arthanareeswaran [6], and Gábor Veréb [1,*]**

## Supplementary material

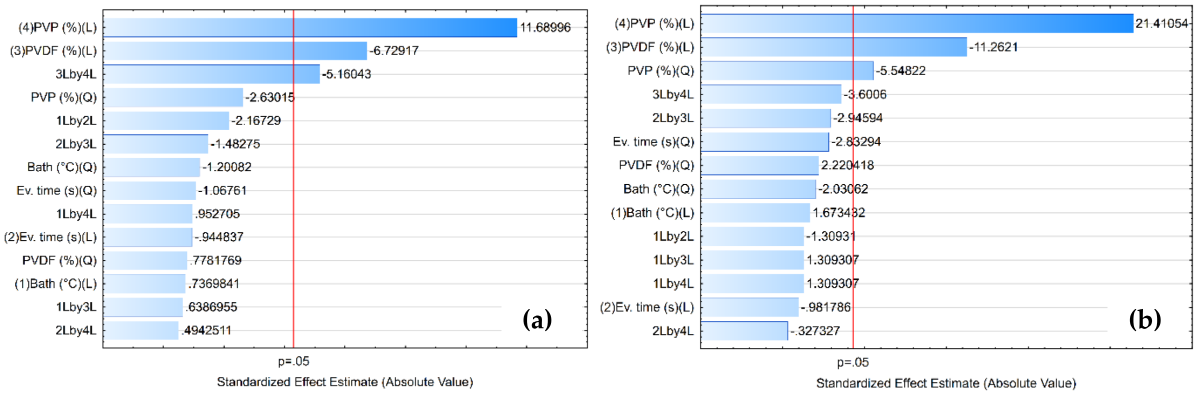


**Figure S1.** Pareto chart for the pure water flux (a) and pore size (b) of the neat-fabricated membranes. Variables with p>0.05 are considered significant

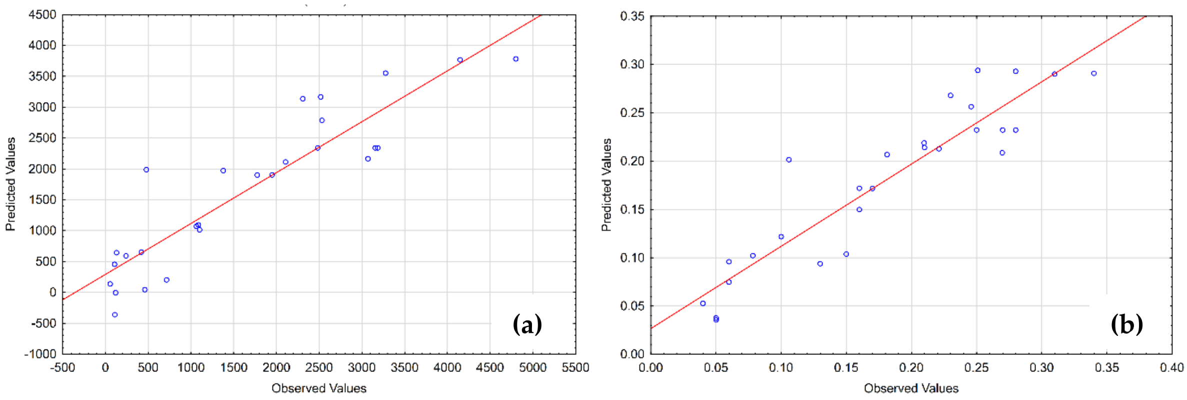


**Figure S2.** Predicted x observed values for the regression analysis of pure water flux (a) and pore size (b) of the neat-fabricated membranes

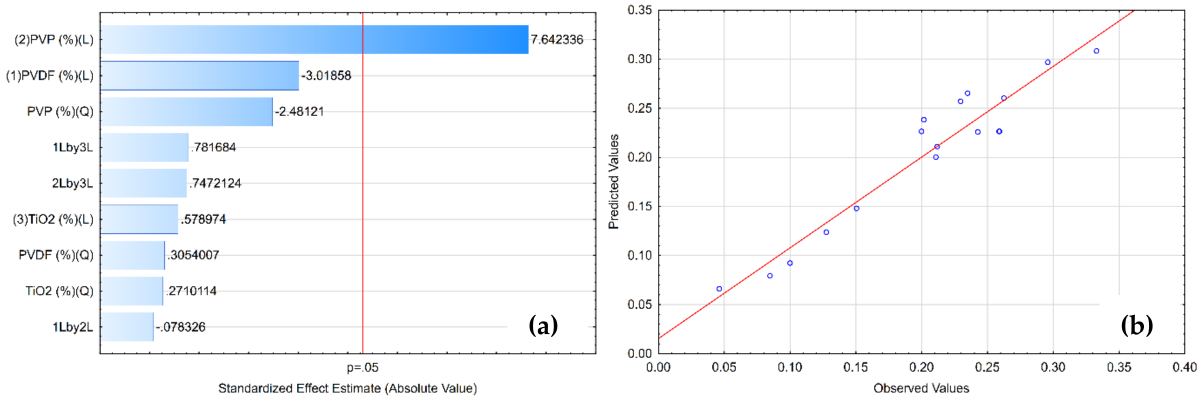


**Figure S3.** Pareto chart (a) and predicted x observed values (b) for the regression analysis of the pore size response of $TiO_2$-modified membranes

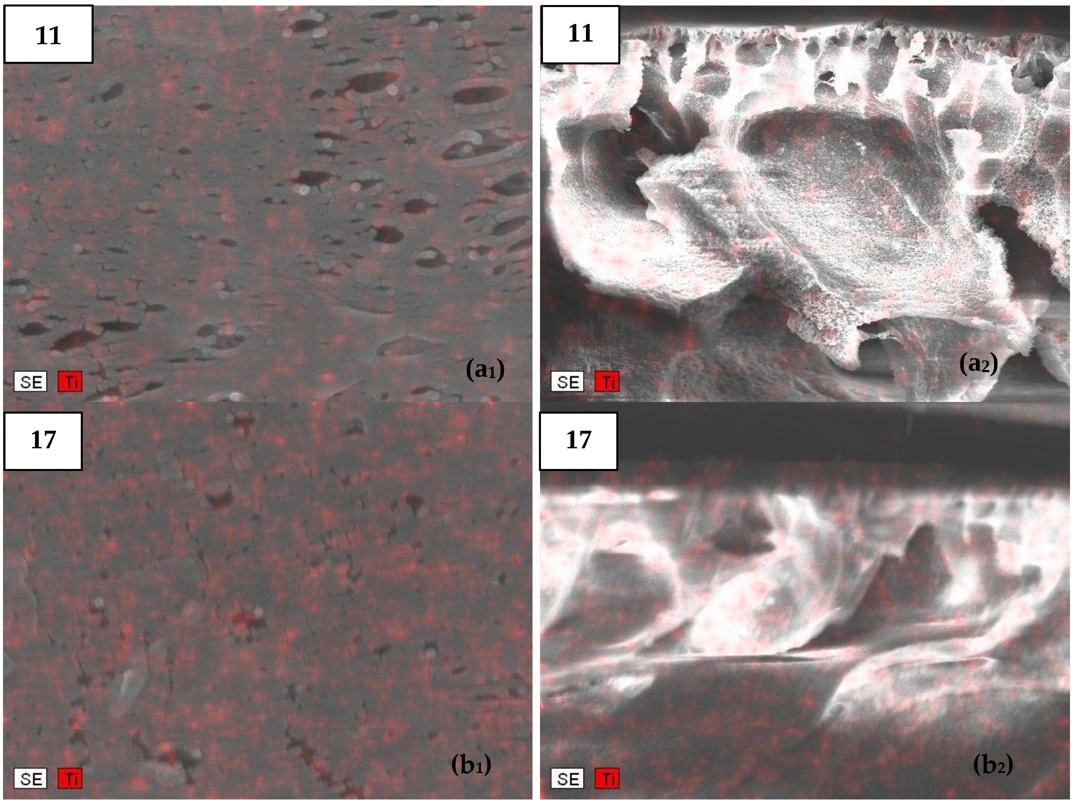


**Figure S4**. SEM and EDX mapping images for membranes 11 (1.5% $TiO_2$) (a) and 17 (3.0 % $TiO_2$) (b), from the top surface ($a_1$, $b_1$) and the cross sections ($a_2$, $b_2$).

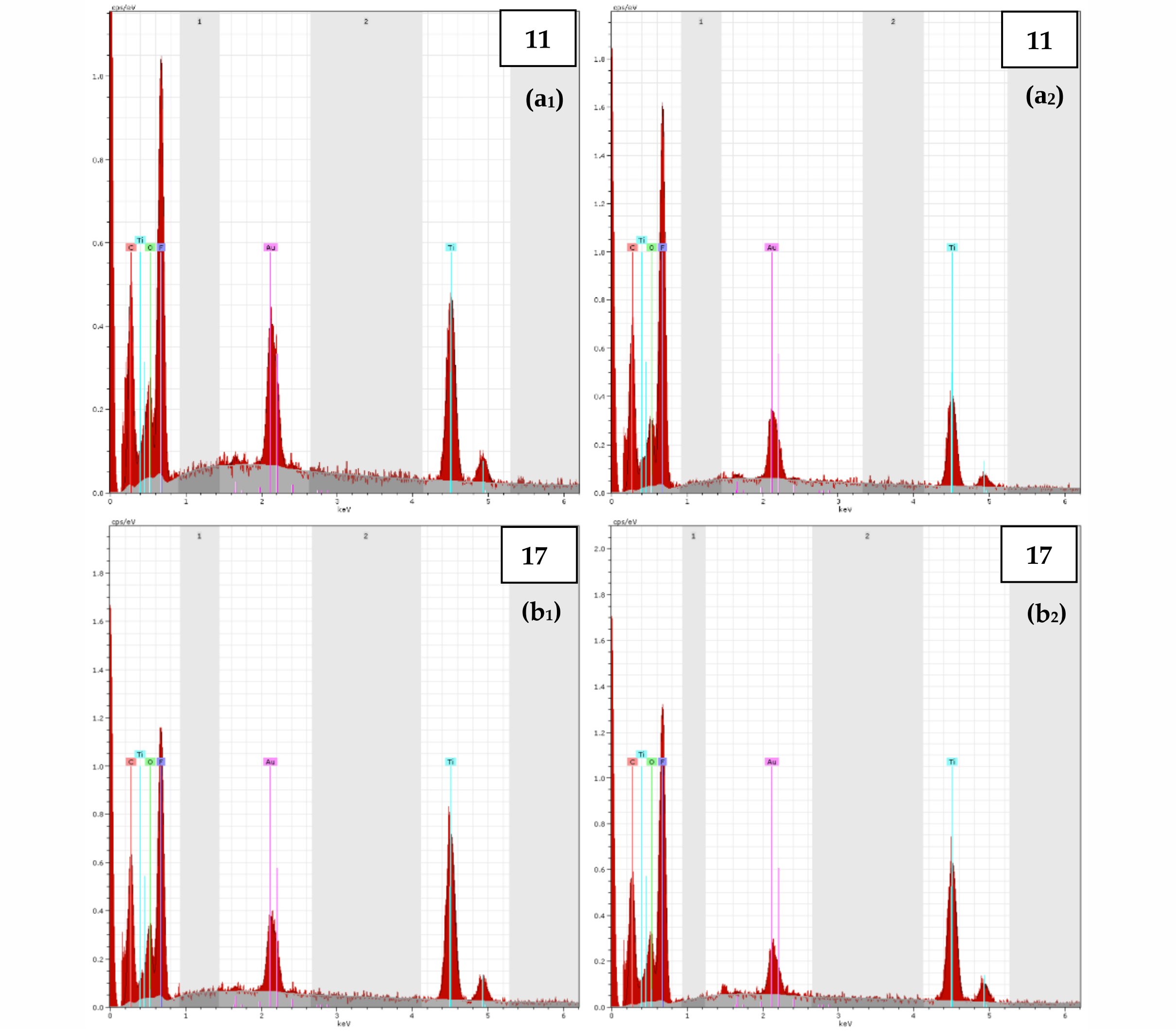


**Figure S5**. Spectra of detected elements of membranes 11 (1.5% $TiO_2$) (a) and 17 (3.0 % $TiO_2$) (b), from the top surface ($a_1$, $b_1$) and the cross sections ($a_2$, $b_2$).

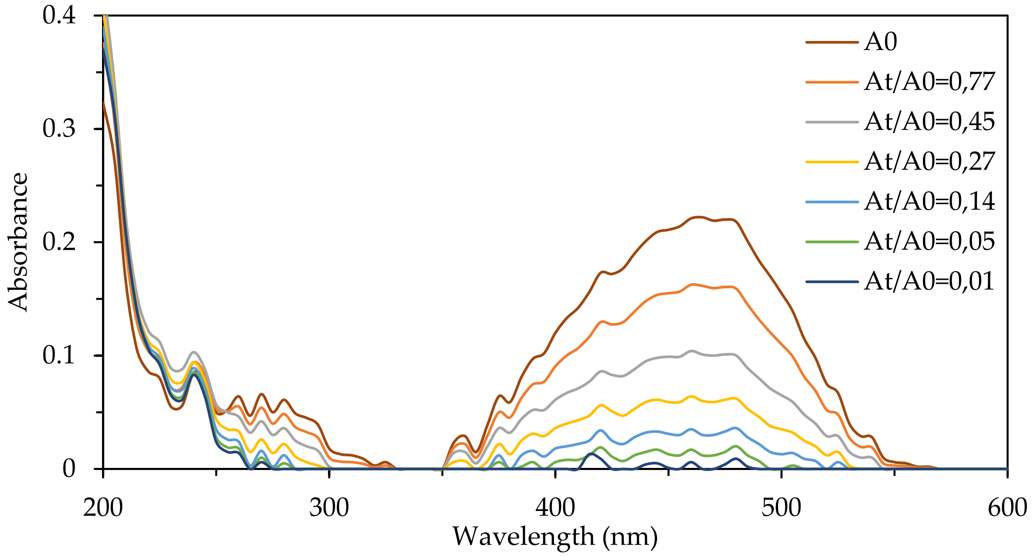


**Figure S6**. Changes in the absorption spectra of the methyl orange solution during photocatalytic decomposition with $TiO_2$. A0 is the spectrum in the beginning of the experiment (t=0) and At is the spectrum in each different time interval (t)

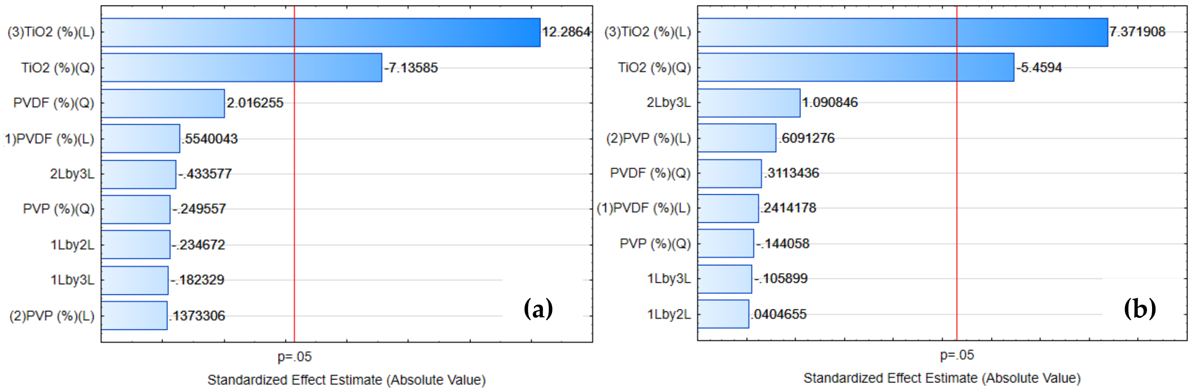


**Figure S7.** Pareto chart for the r response of the natural (a) and acid conditions (b) of the $TiO_2$-modified membranes. Variables with p>0.05 are considered significant

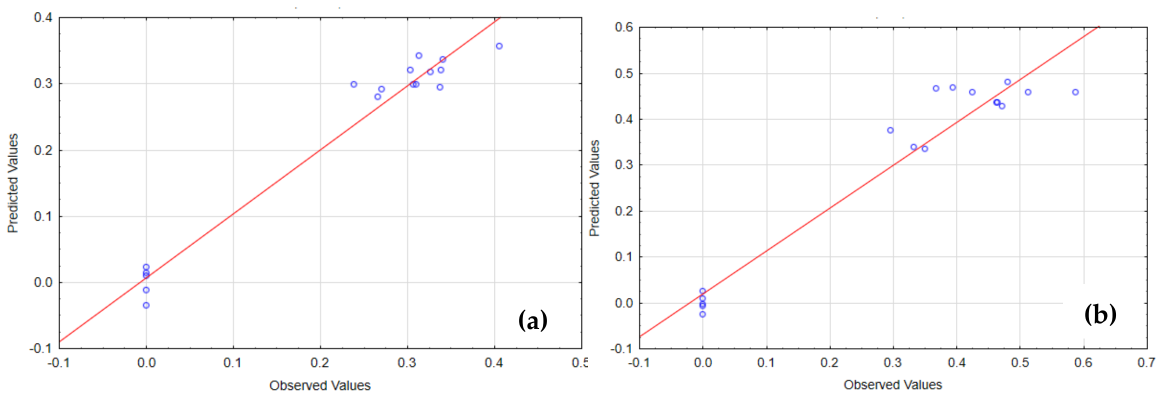


**Figure S8.** Predicted x observed values of r response for the regression analysis of natural (a) and acidic conditions (b) of the $TiO_2$ modified fabricated membranes

**Table S1.** Absorbance values during the photocatalytic experiments using methyl orange in natural pH conditions ($\lambda$=509 nm) and acidic conditions ($\lambda$=466 nm) for the adsorption time and 30 hours UV irradiation. The numbers follow the central composite design and characterization results of the modified membranes (17 experiments: $2^3$ = 8 experiments + 3 (C) center points + 6 (S) star-points).

| Time | Absorbance (natural condition) | | | | | | | | | | | | | | | | |
|---|---|---|---|---|---|---|---|---|---|---|---|---|---|---|---|---|---|
| | **7** | **8** | **3** | **6** | **2** | **4** | **10 C** | **5** | **9 C** | **1** | **11 C** | **12 S** | **13 S** | **14 S** | **15 S** | **16 S** | **17 S** |
| 00 | 0.215 | 0.215 | 0.215 | 0.215 | 0.215 | 0.215 | 0.227 | 0.227 | 0.227 | 0.227 | 0.227 | 0.231 | 0.231 | 0.231 | 0.231 | 0.231 | 0.231 |
| 0 | 0.210 | 0.198 | 0.213 | 0.202 | 0.210 | 0.204 | 0.218 | 0.225 | 0.222 | 0.227 | 0.220 | 0.229 | 0.218 | 0.227 | 0.225 | 0.226 | 0.222 |
| 1 | 0.204 | 0.194 | 0.210 | 0.193 | 0.208 | 0.204 | 0.212 | 0.215 | 0.207 | 0.222 | 0.214 | 0.215 | 0.200 | 0.209 | 0.209 | 0.225 | 0.213 |
| 2 | 0.190 | 0.156 | 0.209 | 0.177 | 0.208 | 0.195 | 0.205 | 0.203 | 0.191 | 0.219 | 0.202 | 0.198 | 0.182 | 0.192 | 0.189 | 0.222 | 0.194 |
| 3 | 0.155 | 0.148 | 0.205 | 0.141 | 0.208 | 0.189 | 0.174 | 0.160 | 0.170 | 0.218 | 0.176 | 0.183 | 0.166 | 0.181 | 0.171 | 0.224 | 0.184 |
| 5 | 0.127 | 0.123 | 0.205 | 0.114 | 0.202 | 0.187 | 0.150 | 0.130 | 0.141 | 0.217 | 0.146 | 0.151 | 0.132 | 0.152 | 0.146 | 0.223 | 0.145 |
| 15 | 0.046 | 0.044 | 0.202 | 0.039 | 0.197 | 0.184 | 0.072 | 0.045 | 0.053 | 0.217 | 0.059 | 0.057 | 0.037 | 0.066 | 0.050 | 0.220 | 0.067 |
| 22 | 0.023 | 0.026 | 0.203 | 0.019 | 0.204 | 0.184 | 0.043 | 0.022 | 0.028 | 0.217 | 0.030 | 0.030 | 0.015 | 0.037 | 0.025 | 0.217 | 0.040 |
| 24 | 0.019 | 0.021 | 0.202 | 0.015 | 0.203 | 0.183 | 0.037 | 0.018 | 0.023 | 0.216 | 0.025 | 0.023 | 0.011 | 0.031 | 0.018 | 0.218 | 0.031 |
| 30 | 0.012 | 0.013 | 0.202 | 0.010 | 0.205 | 0.182 | 0.027 | 0.012 | 0.013 | 0.216 | 0.014 | 0.013 | 0.006 | 0.019 | 0.011 | 0.215 | 0.020 |
| | **Absorbance (acidic condition)** | | | | | | | | | | | | | | | | |
| | **7** | **8** | **3** | **6** | **2** | **4** | **10 C** | **5** | **9 C** | **1** | **11 C** | **12 S** | **13 S** | **14 S** | **15 S** | **16 S** | **17 S** |
| 00 | 0.322 | 0.322 | 0.322 | 0.322 | 0.322 | 0.322 | 0.322 | 0.322 | 0.322 | 0.322 | 0.322 | 0.305 | 0.305 | 0.305 | 0.305 | 0.305 | 0.318 |
| 0 | 0.310 | 0.294 | 0.319 | 0.297 | 0.312 | 0.300 | 0.314 | 0.319 | 0.317 | 0.320 | 0.313 | 0.304 | 0.300 | 0.300 | 0.298 | 0.299 | 0.320 |
| 1 | 0.285 | 0.260 | 0.316 | 0.269 | 0.297 | 0.293 | 0.279 | 0.288 | 0.281 | 0.312 | 0.280 | 0.267 | 0.258 | 0.258 | 0.261 | 0.298 | 0.299 |
| 2 | 0.243 | 0.200 | 0.313 | 0.234 | 0.304 | 0.291 | 0.228 | 0.243 | 0.236 | 0.316 | 0.238 | 0.235 | 0.227 | 0.228 | 0.228 | 0.294 | 0.264 |
| 3 | 0.218 | 0.195 | 0.317 | 0.221 | 0.303 | 0.292 | 0.191 | 0.226 | 0.203 | 0.315 | 0.199 | 0.206 | 0.199 | 0.205 | 0.202 | 0.296 | 0.252 |
| 5 | 0.163 | 0.147 | 0.317 | 0.180 | 0.301 | 0.290 | 0.133 | 0.183 | 0.157 | 0.315 | 0.145 | 0.166 | 0.154 | 0.160 | 0.168 | 0.295 | 0.212 |
| 15 | 0.050 | 0.045 | 0.319 | 0.073 | 0.292 | 0.278 | 0.023 | 0.060 | 0.033 | 0.312 | 0.028 | 0.049 | 0.034 | 0.037 | 0.054 | 0.292 | 0.084 |
| 22 | 0.014 | 0.013 | 0.319 | 0.032 | 0.285 | 0.275 | 0.006 | 0.030 | 0.017 | 0.316 | 0.010 | 0.022 | 0.012 | 0.014 | 0.023 | 0.288 | 0.043 |
| 24 | 0.011 | 0.010 | 0.319 | 0.026 | 0.284 | 0.274 | 0.004 | 0.024 | 0.013 | 0.316 | 0.007 | 0.016 | 0.009 | 0.010 | 0.021 | 0.290 | 0.038 |
| 30 | 0.004 | 0.004 | 0.319 | 0.013 | 0.278 | 0.271 | 0.001 | 0.013 | 0.007 | 0.318 | 0.003 | 0.008 | 0.004 | 0.004 | 0.010 | 0.286 | 0.022 |
